# Analysis and Optimization of the Milling Performance of an Industry-Scale VSM via Numerical Simulations

**DOI:** 10.3390/ma16134712

**Published:** 2023-06-29

**Authors:** Chengguang Tong, Zuobing Chen, Chang Liu, Qiang Xie

**Affiliations:** 1 School of Mechanical and Electronic Engineering, Wuhan University of Technology, Luoshi Road, Wuhan 430070, China; 250132@whut.edu.cn (C.T.); zbchen01@126.com (Z.C.); 2 Hefei Zhongya Building Material Equipment Co., Ltd., Hefei 230601, China; liu290155@163.com

**Keywords:** industry-scale vertical stirred mill, grinding performance, discrete element method, response surface method, multi-objective optimization

## Abstract

Vertical stirred mills (VSM) are widely used for powder processing in many situations like mechanical alloying preparation and raw material crushing and shaping. Many structural and operational parameters like stirrer helix angle and rotating speed have great significance on VSM performance, especially in a large industry-scale situation. Therefore, it becomes essential to investigate these parameters systematically to obtain high energy efficiency and good product quality. In this work, the discrete element method (DEM) was used to examine the effects of stirrer helix angle (α), stirrer diameter (*d*), and rotating speed (*n*) on the grinding performance in an industrial VSM, and then the response surface method (RSM) was employed for multi-objective optimization in the VSM. It is found that a media vortex phenomenon may happen near the stirring shaft. The media collisions are significantly influenced by α, *d*, and *n*. Through multi-objective optimization design (MOD), the power consumption (*P*) of the stirrer reduced by 8.09%. The media collision energy (*E*) increased by 9.53%. The energy conversion rate (*R*) rises by 20.70%. The collision intensity and frequency are both improved. This optimization method can help determine good operating parameters based on certain structures.

## 1. Introduction

Usually, it is common to apply a vertical stirred mill (VSM) in process industries handling various kinds of powders, especially at ultra-fine scale. The main component of a VSM includes a stirrer and a cylinder [1,2,3,4]. The stirrer rotates, moving media balls and grinding materials through multiple mechanisms like shearing, extrusion and friction [2,5]. As a stirred media mill, the VSMs have unique properties in many aspects like energy intensity and scale-up [6]. For example, compared to conventional ball mills, they have many advantages like high efficiency, low noise, and good powder shapes. Therefore, they are commonly employed in numerous scenarios like mechanical milling [6], building material preparation, cement production, mineral separation, and so on [7,8,9,10].

The milling and grinding performance as well as energy consumption in a VSM are always paid a lot of attention. In China, according to statistics, the output value of the national powder industry has accounted for more than 50% of the total output value of the first and second industries [11]. The development of powder preparation technology will promote many industries and have a noteworthy impact on the overall national economic level [12,13]. With the increasing demand for ultrafine powder in the market [14], larger-scale VSMs will be needed [15,16]. Therefore, it is necessary to run the VSM with good performance and low energy. The VSM performance is affected by many factors like the structures, material properties, and operating parameters, which together influence the flows and interactions between the media balls and the materials [17,18,19,20].

This topic received numerous investigations via multiple research methods. For example, Rhymer et al. investigated the effects of changing the particle–particle restitution and sliding friction coefficients of grinding media. It is considered that the material as a lubricant can be assumed to change the friction coefficient of the grinding media to simplify this factor [21]. The feasibility of their work is verified. Daraio et al. took the number of collisions, energy dissipation, and medium velocity as indicators to determine the grinding process conditions and material characteristics [22]. The collision frequency is defined as the number of collisions per second that take place in the mill, whereas the power dissipation is defined as the power lost during the media motion through collisions, due to the normal and tangential dumping forces. It was found that the eddy current medium in the center of the agitator moved towards the wall of the vessel, and the grinding effect in this area was poor, owing to the small velocity gradient of the medium. Oliveira et al. used the DEM method to predict VSM power and found that the deviation between prediction and measurement was close, demonstrating the ability of the contact parameters in compensating for the absence of materials in the simulations [23]. They also analyzed in their cases the simulated contribution of the normal and tangential energy, and the latter was responsible for about 68% of energy dissipation in the simulated mill. The increase in stirrer tip speed resulted in not only power growth but also the frequency of both ball–ball and ball–geometry collisions. Through experiments, Danielle Rocha et al. found that the largest grinding media will promote a higher breakage rate of larger particles, while the smaller media is preferred for finer particles [24]. It is necessary to increase the power input to raise the crushing rate. A finer product size is obtained when the mill operates at a higher stirrer speed. Moreover, Maruf Hasan et al. revealed that higher mill tip speed, lower solids concentration, and smaller grinding media size could generate finer particle size distribution in a batch mill [25]. Elizma Ford et al. explored the effect on power draw and grinding performance when adding a shell liner to a vertical fluidized stirred media mill [26]. Alessandro et al. studied the effects of rotational speed, filling rate, medium size, and agitator diameter on the grinding performance of crushed powder [27]. David et al. researched the influence of materials and found that the increase of medium friction increases power consumption and energy transfer coefficient [28].

Analyzing these studies, some relevant conclusions can be drawn. First, it is recognized that it is feasible to compensate for the lubrication effect of materials by changing the friction coefficient of the medium. Second, it is acceptable to use the DEM method to study the deviation between VSM and experimental results, and simulation can save a lot of time and cost. Third, the vortex medium near the stirring shaft has a small velocity gradient, which leads to the failure of shear grinding and energy loss. Fourth, the most important factors affecting the crushing rate are speed, medium size, agitator diameter, filling rate, agitator form, equipment material, and grinding time. Fifth, there is a direct relationship between medium collision and material crushing effect. These studies have comprehensively investigated the grinding law in principally laboratory-scale VSMs from all aspects, focusing mainly on the effects of a single factor, respectively, and the crushing mechanisms. Their research achievements may also be useful for large industry-scale VSMs to some degree. Nevertheless, few investigations have been carried out concentrating on large industry-scale VSMs, and inevitably there are differences in various scales. It is always of great significance how to determine the optimal related parameters and obtain the lowest power consumption, while simultaneously reaching the best grinding performance.

With the development of computing and modeling techniques, simulating large VSMs directly via the discrete element method has become feasible. Therefore, based on previous studies and achievements, this work focuses on the granular flows and grinding behaviors in a large industry-scale VSM using DEM simulations aiming at a deep understanding of equipment performance. A relatively comprehensive set of indicators was proposed to describe the VSM performance. Several corresponding values were analyzed. Moreover, a multi-objective optimization design (MOD) based on the response surface method (RSM) was carried out and finally, the optimized parameters for the VSM were obtained.

The rest of this paper is arranged as follows. Section 2 describes of numerical calculation model and method; Section 3 introduces the establishment of the evaluating indicators and methodology; Section 4 demonstrates the promising experimental results, followed by concluding remarks in Section 5.

## 2. Numerical Model Description

### 2.1. Governing Equations

In a VSM granular system, the grinding balls could be treated as discrete elements and their movements follow Newton’s law. All motion parameters for each ball, like position and velocity, translational and rotational, could be tracked over time. To simulate the moving behaviors of all the balls, the commercial software package EDEM 2020 has been used in this work [29]. The governing equations of the transnational and rotational motion for any ball *i* are, respectively, expressed as

(1) $$m_{i}\frac{dv_{i}}{dt}=m_{i}g+\sum_{i}\left(F_{n,ij}+F_{t,ij}\right)$$

(2) $$I_{i}\frac{d\omega_{i}}{dt}=-\mu_{r,ij}F_{ij}R_{i}\vec{\omega_{i}}+\sum_{i}R_{i}\times F_{t,ij}$$

where *m*, *v*, *w*, and *I*, are the mass, velocity, angular velocity, and rotational inertia, respectively, $F_{n,ji}$, and $F_{t,ij}$ are the normal and tangential forces between balls *i* and *j*, respectively, *g* is the acceleration of gravity, $v_{i}$ is the moving velocity of ball *i*, $R_{i}$ is the radius of ball *i*, and *t* denotes time.

**Figure 1:**
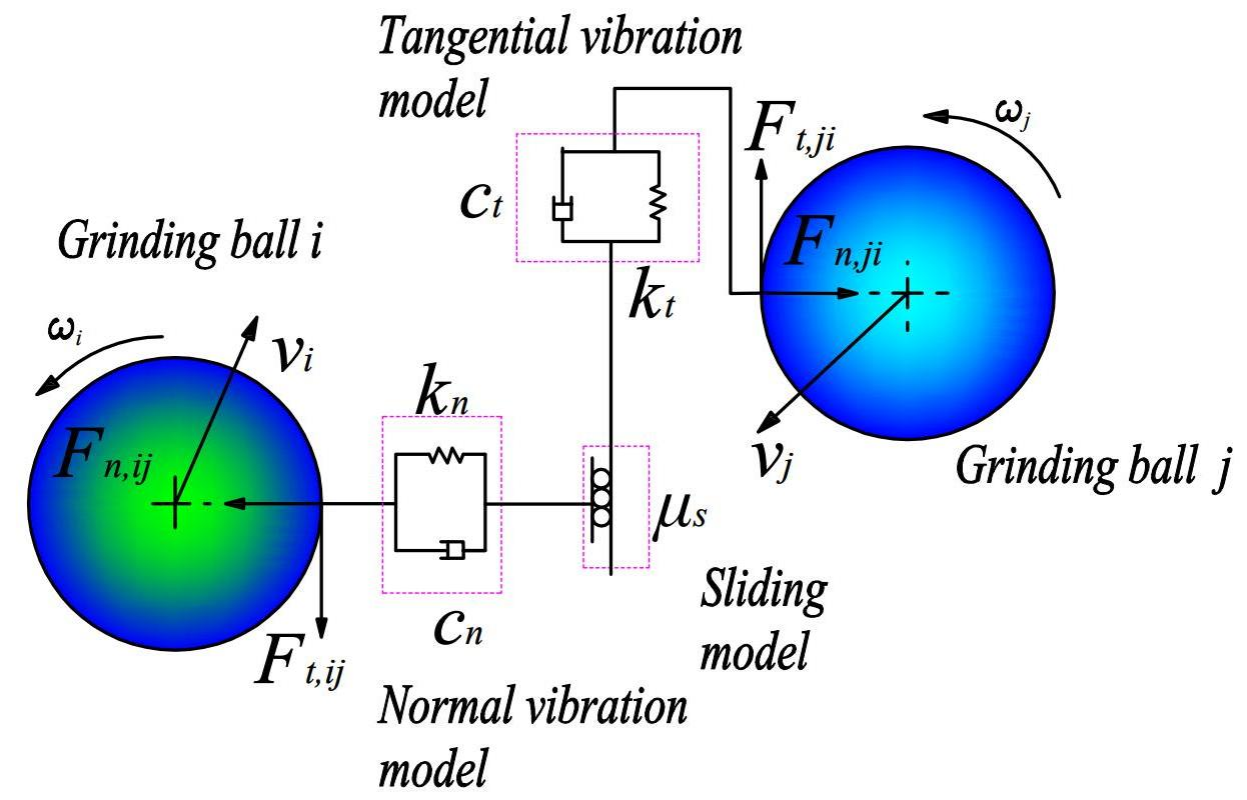
Schematic diagram of the contact model for balls *i* and *j*.

The contacting interactions between balls *i* and *j* in a large dry VSM focused on in this work could be described according to the normal Hertz–Mindlin model (Figure 1) [29,30].

The normal and tangential forces between balls *i* and *j* could be expressed, respectively, by

(3) $$F_{n,ij}=-\frac{4}{3}E^{*}\sqrt{R^{*}}\delta^{\frac{3}{2}}+\sqrt{\frac{20}{3}}\beta {\left(m^{*}E^{*}\sqrt{R^{*}\delta_{n}}\right)}^{\frac{1}{2}}v_{n,ij}$$

(4) $$F_{t,ij}=\min\left[\mu_{s,ij}F_{n,ij},8G^{*}\delta_{t}\sqrt{R^{*}\delta_{n}}+\sqrt{\frac{80}{3}}\beta {\left(m^{*}G^{*}\sqrt{R^{*}\delta_{n}}\right)}^{\frac{1}{2}}v_{t,ij}\right]$$

where $\delta_{n}$ and $\delta_{t}$ are the normal and tangential overlaps of the two balls, respectively. $v_{n,ij}$ and $v_{t,ij}$ are the relatively normal and tangential velocities between the two balls, respectively. $\mu_{s,ij}$ is the static friction coefficient between the two balls. $E^{*}$, $R^{*}$, $G^{*}$, and $m^{*}$ are the equivalent elastic modulus, equivalent contact radius, equivalent shear modulus, and equivalent mass of the two balls, respectively, and they are given by

(5) $$E^{*}=\frac{E_{i}E_{j}}{E_{i}+E_{j}}$$

(6) $$m^{*}=\frac{m_{i}m_{j}}{m_{i}+m_{j}}$$

(7) $$R^{*}=\frac{R_{i}R_{j}}{R_{i}+R_{j}}$$

(8) $$G^{*}=\frac{1-u_{i}^{2}}{G_{i}}+\frac{1-u_{j}^{2}}{G_{j}}$$

where $E_{k}$, $m^{*}$, $G^{*}$, $R^{*}$, and $u_{k}$(k=i,j) denote Young’s modulus, mass, shear modulus, radius, and Poisson’s ratio of balls *i* and *j*, respectively.

### 2.2. Geometry Model

**Figure 2:**
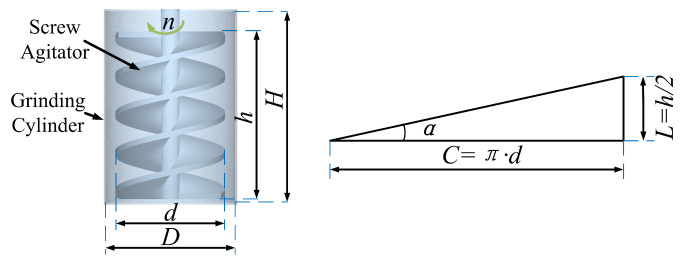
The geometry model of the vertical stirred mill (α is stirrer helix angle).

The VSM as shown in Figure 2 is mainly composed of two parts: the screw agitator and the grinding cylinder [31]. Table 1 gives the primary structural parameters focused on in this work. The grinding media are corundum balls of two sizes , ϕ15 mm and ϕ22 mm.

### 2.3. Simulation Conditions

The contact parameters used in the present work were generally considered and determined from the references [21,22,23,27]. The simulating parameters are shown in Table 2 below. The stable operation data are collected and analyzed to ensure the accuracy of the results. There are about 3.39 million grinding media in the mill. Considering the large number of particles, the simulation results like the position, speed, collision, and other information of grinding media during a small time step are automatically saved to avoid abnormal interruption. After starting, it takes about 8 s of simulation time to charge the grinding media. Therefore, the computational results were obtained after the media was stabilized to reduce error.

The stirrer helix angle (α), stirrer diameter (*d*), and rotating speed (*n*) are used as three influencing factors. For the parameter determination of the variables of RSM, α corresponds to the ratio of lead to the diameter of the agitator. When the ratio is lower than 0.7 or higher than 0.9, it is found that the grinding efficiency will drop sharply, which is not meaningful for research and thus not shown. Similarly, *d* affects the gap size between the agitator and the cylinder wall, and *n* affects the edge line speed of the agitator. After a series of computational tests, the optimal value matching will not be generated if the parameters exceed a certain range. Consequently, three levels are designed for each factor, and they are listed in Table 3.

### 2.4. Model Verification

**Figure 3:**
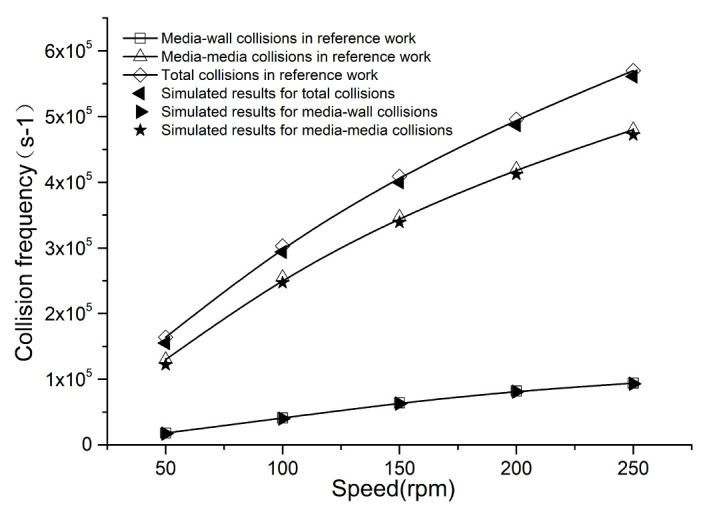
The collision frequency as speed in comparison with reference [22].

**Figure 4:**
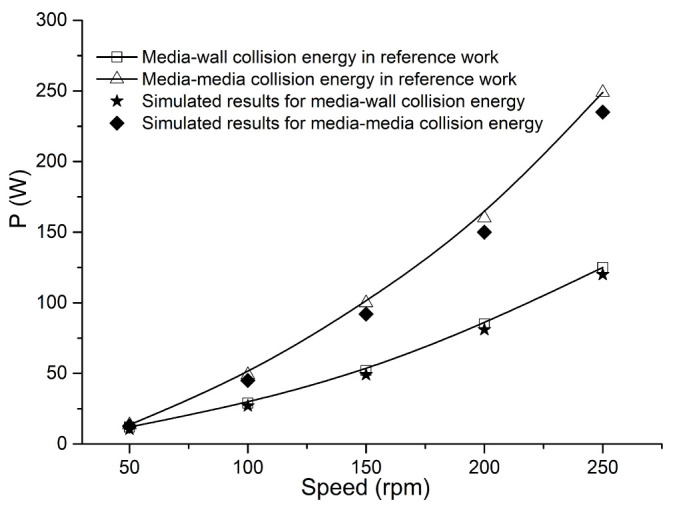
The power dissipation as speed in comparison with reference [22].

For the sake of completeness, a separate simulation has been carried out to verify the numerical model employed in this work, even though it has been examined in several previous studies [32,33]. The numerical model in this paper is used to calculate the same stirring mill in the reference [22] and make a comparative analysis. The simulation conditions like the media ball diameter, ball density, Young’s modulus, and Poisson’s ratio are unchanged. More details can be found in the work of the authors in [22]. The open-source software LIGGGHTS was employed in the reference, which is the biggest difference. Through simulations and data analysis, the compared results are shown in Figure 3 and Figure 4, giving the collision counts and energies under different stirring speeds. Good agreements could be found between our simulations and the reference work, including the changing trends and values on the power and collision frequency. Their relative errors are both within 5%. In this case, we think that the numerical model applied in our work is reasonable. Therefore, the follow-up simulations could be continued based on the verified computing model.

## 3. Evaluation Indicators and Optimization Method

### 3.1. Collision Performance Indicators

The tangential collision force ($F_{T}$), normal collision force ($F_{N}$), collision frequency (*f*), power (*P*), collision energy (*E*), and energy conversion rate (*R*) are six important indexes for the VSM performance.

(1) The tangential and normal collision forces are defined as
  (9) $$F=\frac{F_{\phi 15-\phi 15}+F_{\phi 15-\phi 22}+F_{\phi 22-\phi 22}}{3}$$
  where *F* is average collision force and the $F_{\phi 15-\phi 15}$, $F_{\phi 15-\phi 22}$, $F_{\phi 22-\phi 22}$ are, respectively, average collision force between ϕ15 mm media, ϕ15 mm and ϕ22 mm media, ϕ22 mm media.

(2) Due to the saving time interval Δt, the collision frequency is defined as
  (10) $$n_{i}=n_{\phi 15-\phi 15}+n_{\phi 15-\phi 22}+n_{\phi 22-\phi 22}$$
  (11) $$\bar{n}=\sum_{i=1}^{N_{i}}n_{i}/N_{t}^{T}$$
  (12) $$f=\bar{n}/\Delta t$$
  where $n_{i}$ is the total number of collisions of media in Δt, $n_{\phi 15-\phi 15}$, $n_{\phi 15-\phi 22}$, $n_{\phi 22-\phi 22}$ are number of collisions between ϕ15 mm media, ϕ15 mm and ϕ22 mm media, and ϕ22 mm media, respectively. $\bar{n}$ is average total number of collisions, $N_{t}$ is number of nodes saved per Δt data, *f* is collision frequency [22].

(3) The power is defined as
  (13) $$\bar{T}=\sum_{i=1}^{N_{i}}T_{i}/N_{t}$$
  (14) $$P=\bar{T}\cdot n/9550$$
  where $\bar{T}$ is average torque at different moments; $T_{i}$ is stirrer torque at each moment; $N_{t}$ is the number of nodes per Δt data saving; *P* is stirrer power; *n* is stirrer speed; 9550 is a constant on the relationship between power, rotary speed, and torque [34].

(4) The collision energy is defined as
  (15) $$E_{i}=E_{\phi 15-\phi 15}+E_{\phi 15-\psi 22}+E_{\phi 22-\phi 22}$$
  (16) $$\bar{E}=\sum_{i=1}^{N_{i}}E_{i}/N_{t}$$
  (17) $$E_{i}=E_{\phi 15-\phi 15}+E_{\phi 15-\psi 22}+E_{\phi 22-\phi 22}$$
  where $E_{i}$ is the total collision energy loss of dielectric sphere in Δt, $E_{\phi 15-\phi 15}$, $E_{\phi 15-\phi 22}$, $E_{\phi 22-\phi 22}$ are collision energy loss between ϕ15 mm media, ϕ15 mm and ϕ22 mm media, ϕ22 mm media, respectively. $\bar{E}$ is average total collision energy loss, $N_{t}$ is number of nodes saved per Δt data, *E* is collision energy loss per unit time.

(5) The energy conversion rate is defined as
  (18) R=E/P
  where *R* is the energy conversion rate, *E* is the total collision energy of the grinding media within the unit, and *P* is the power applied to the stirrer.

### 3.2. Response Surface Methodology Prediction Model

The Response Surface Methodology (RSM) is widely applied for multi-objective optimizations [35,36]. According to the principle of multiple nonlinear regression, the predictive model of the media collision characteristics could be established based on RSM results, and the prediction model in the coding space can be expressed as [34].

(19) $$y=f\left(x_{1},x_{2},x_{3}\right)=b_{0}+\sum_{m=1}^{3}b_{m}x_{m}+\sum_{n<m}^{3}b_{nm}x_{n}x_{m}+\sum_{m=1}^{3}b_{mm}x_{m}^{2}$$

where *y* is the response, $x_{1}$ is α in the coding space, $x_{2}$ is *d* in the coding space, $x_{3}$ is *n* in the coding space, and *b* is the regression coefficient in the coding space, which can be described as follows:

(20) $$b_{0}=1/R\sum_{S=1}^{R}y_{s}$$

(21) $$b_{m}=\sum_{S=1}^{R}x_{sm}y_{s}/\sum_{S=1}^{R}x_{sm}^{2}$$

(22) $$b_{xm}=\sum_{S=1}^{R}x_{sm}x_{sm}y_{s}/\sum_{S=1}^{R}{\left(x_{sm}x_{sm}\right)}^{2}$$

(23) $$b_{mm}=\sum_{S=1}^{R}\left(x_{sm}^{2}-1/R\sum_{S=1}^{R}x_{sm}^{2}\right)y_{s}/\sum_{S=1}^{R}\left(x_{sm}^{2}-1/R\sum_{S=1}^{R}x_{sm}^{2}\right)$$

where *R* is the number of simulations. When the prediction model in the coding space is obtained, the coding Equation (24) is introduced into Equation (19) to obtain the predictive model (Equation (25)) in the natural space.

(24) $$x_{m}=\left(x_{j}-x_{0m}\right)/\Delta_{m}$$

where $x_{0m}$ is zero level of the influence factor, and $\Delta_{m}$ is level increment of the influence factor.

(25) $$y=f\left(x_{1},x_{2},x_{3}\right)=\beta_{0}+\sum_{m=1}^{4}\beta_{m}x_{m}+\sum_{n<m}\beta_{nm}x_{n}x_{m}+\sum_{m=1}^{4}\beta_{mm}x_{j}^{2}$$

where *x* is influence factor in natural space, and β− regression coefficient in the natural space.

## 4. Results and Discussions

### 4.1. Media Motion Analysis

**Figure 5:**
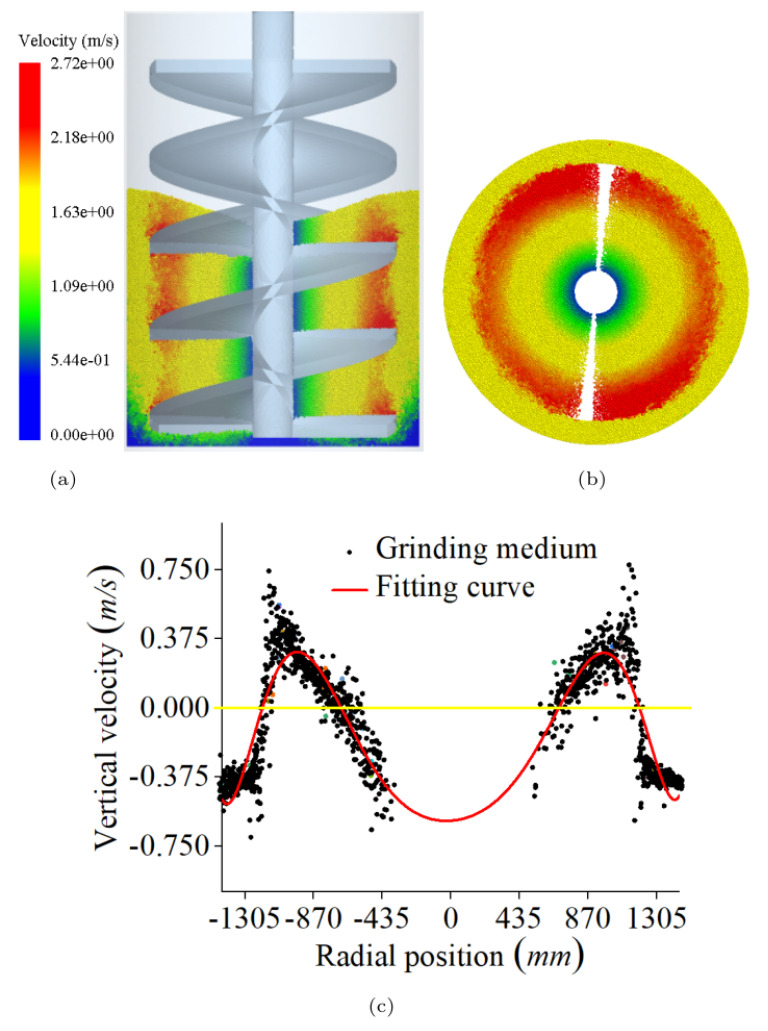
Media velocity distribution. (**a**) Longitudinal center section, (**b**) Horizontal transverse section at 1200 mm height, (**c**) Media vertical velocity distribution along the radial direction at 1000 mm height.

Figure 5a,b depict the simulation results based on the original structural parameters in Table 1 and Table 2. As shown in Figure 5c, the vertical velocity and radial position of each media correspond. It shows the red line below the yellow line in the radial position −750 mm to 750 mm, indicating that the media is moving downward due to the low tangential velocity, which cannot generate enough centripetal force to balance the stirrer force and its own gravity. Part of the medium formed a vortex phenomenon, resulting in the area of the medium being almost stationary, and unable to produce grinding shear effect on the material. Therefore, a larger vortex area will lead to ineffective grinding, which is harmful to the VSM.

**Figure 6:**
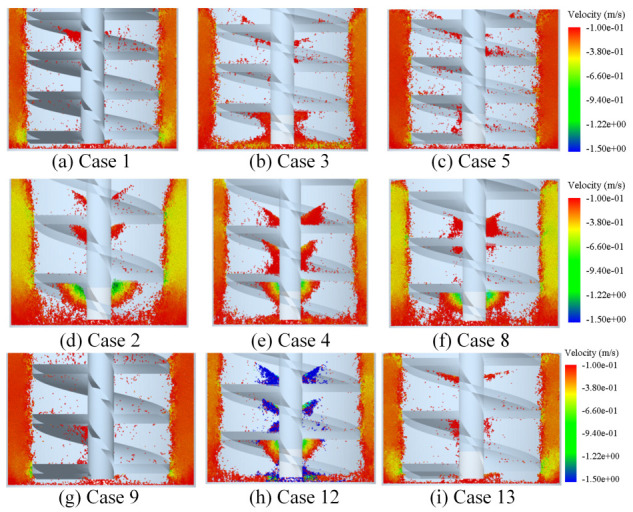
Distribution of nine representative groups of media with vertical velocity downward at t = 15 s.

The amount of vortex media near the stirring shaft varies with α, *d*, and *n*. However, the fluctuation of *n* in the studied range turns out to have little effect on the eddy current phenomenon and has therefore been ignored. In addition, analysis of the factors α and *d* are focused on. The conditions of all simulation cases can be found in Table 4. Nine groups of simulation results are selected and displayed in Figure 6. Only the downward moving media are retained, with the minus sign “-” indicating the direction of vertical velocity is downward. In each row, the three cases have the same α with altered *d*, while the three cases in each column have the unchanged *d* and different α. Compared with the top row, it is found that Case 3 with the largest *d* forms the most eddy media. The next two rows have the same pattern and trend, indicating that vortex media is easier to emerge with a rising *d*. Comparing the three cases in the leftmost column, it is found that Case 2 with the largest α has the most eddy media. Similarly, the next two columns show the same changed laws, indicating that an increasing α could enhance the formation of vortex media.

### 4.2. Collision Characteristics Analysis

Based on the Box–Behnken method [35,37], the experiment schemes have been designed. There are 17 cases in total. All simulations have been carried out like the one discussed before. Through a series of analyses, results of interest, representing grinding performance and energy consumption, have been acquired. For simplicity, the designed experiment schemes and the simulated results, i.e., the response values, are presented in Table 4. These data occupy an important place in this work. In addition, they are the basis for later analysis.

With the RSM results, Analysis of Variance (ANOVA) is used in this work to investigate the impact degree of α, *d*, and *n* on the media collision characteristics, and the results are presented in Table 5, Table 6, Table 7, Table 8, Table 9 and Table 10. With a significant level of 0.0001, *n* has the greatest influence on $F_{T}$, and *d* has the greatest influence on $F_{N}$ with a significance level of 0.0013. Furthermore, *n* is one of the most important factors for *f*, *P*, and *R*, while α has the most influence on *E*.

**Figure 7:**
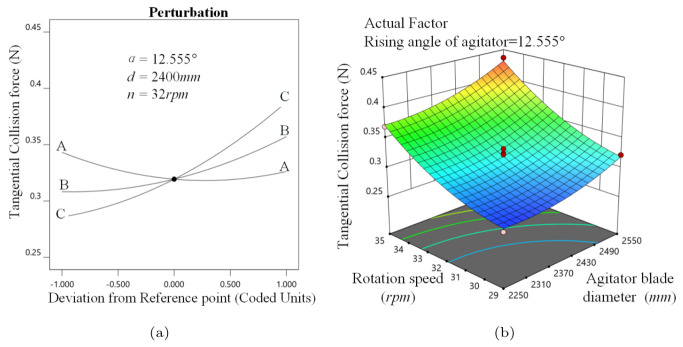
Tangential, collision force analysis, (**a**) α, *d*, *n* perturbation effect on $F_{T}$, in which A, B and C stand for respectively the stirrer helix angle, stirrer diameter and rotary speed; (**b**) d−n interaction on $F_{T}$.

Figure 7a illustrates that $F_{T}$ increases continuously with *d* and *n*, with *n* having more influence because *n* plays a dominant role in the intensity of media movement. Furthermore, increasing α reduces $F_{T}$.

As shown in Figure 7b, when 2490 mm ≤ *d* ≤ 2550 mm and 32 rpm ≤ *n* ≤ 35 rpm, the response surface becomes steep, revealing that a larger *d* has a stronger influence on $F_{T}$ when the stirrer mill is running at high speed. The response surface is gentle when 2250 mm ≤ *d* ≤ 2490 mm and 29 rpm ≤ *n* ≤ 32 rpm, inferring that *d* has less influence on $F_{T}$ when the stirrer mill is running at low speed.

**Figure 8:**
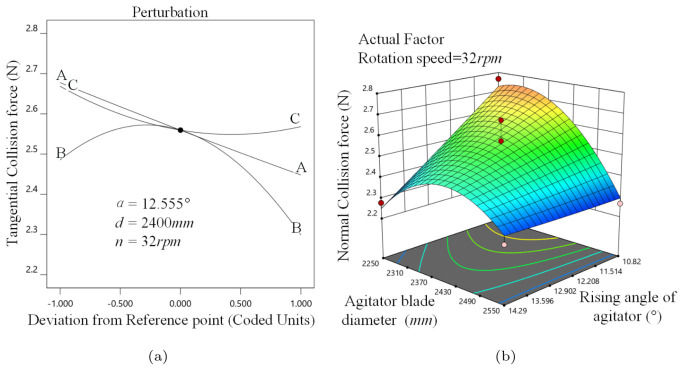
Normal collision force analysis, (**a**) α, *d*, *n* perturbation effect on $F_{N}$, (**b**) α−n interaction on $F_{N}$.

α determines the direction of the stirrer force. Figure 8a shows that $F_{N}$ decreases linearly as α increases. $F_{N}$ rises and then decreases when *d* enlarges. This is because boosting *d* strengthens the perturbation effect initially, and as it keeps increasing it causes the movement of almost all media to be consistent. $F_{N}$ reduces and then smooths as *n* enhances because *n* is primarily reflected in the change in tangential linear velocity, and the influence on $F_{N}$ declines as *n* increases.

As shown in Figure 8b, the response surface becomes sharp when 2250 mm ≤*d*≤ 2370 mm and $12.902^{\circ }$≤α≤$14.29^{\circ }$, because the ball size change has significant influence on $F_{N}$ when *d* is small. When α is small, the effect of *d* grows.

**Figure 9:**
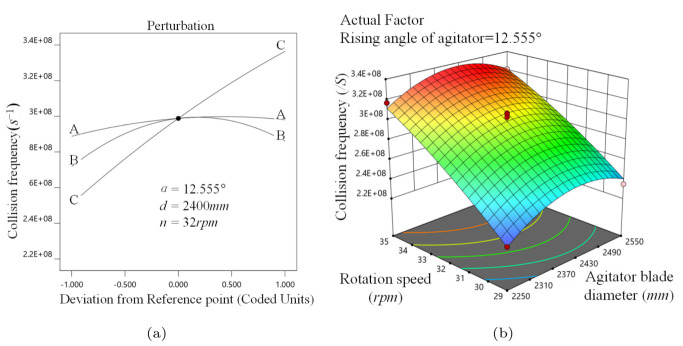
Collision frequency analysis, (**a**) α, *d*, *n* perturbation effect on *f*, (**b**) d−n interaction on *f*.

Figure 9a implies that *f* holds steady with α, implying that α has little influence on media collision motion. When α is $12.555^{\circ }$, the variation of the response surface along the *n* direction is sharper, inferring that the effect of *n* is stronger, as illustrated in Figure 9b. The response surface is steepest when 29 rpm ≤*n*≤ 30 rpm and 2250 mm ≤*d*≤ 2310 mm, implying that *d* has more influence on *f* when the stirred mill is at a relatively low speed.

**Figure 10:**
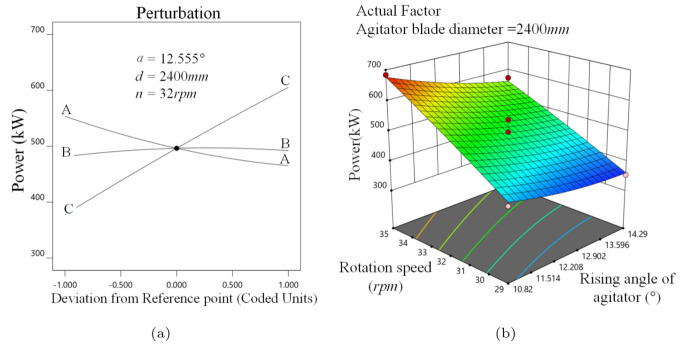
Power analysis, (**a**) α, *d*,*n* perturbation effect on *P*, (**b**) α−n interaction on *P*.

As expected, *P* decreases with α, as shown in Figure 10a. This is due to a smaller α resulting in a less effective stirring volume of media. However, *P* increases slightly as *d* enlarges. That is maybe because a larger *d* results in a greater area of force on the stirrer. In addition, *P* increases linearly with *n*, which could also be known from the Equation (21).

As shown in Figure 10b, the alteration in the response surface along *n* direction is steeper when *d* is 2400 mm, indicating that *n* has a greater effect on *P*. From the bottom curve bar of the response surface, it is clear that a $3.47^{\circ }$ change has the same effect as a 1 rpm change in the *n*.

**Figure 11:**
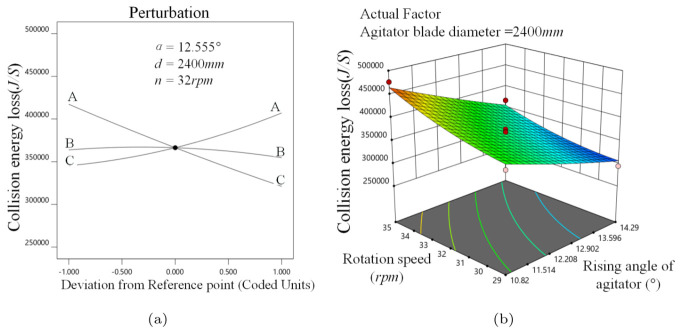
Collision energy analysis, (**a**) α, *d*, *n* perturbation effect on *E*, (**b**) α−n interaction on *E*.

Figure 11a reveals that *E* decreases gradually as α increases, because of the greater vortex media. Moreover, *d* has a lesser effect on *E*. However, *E* grows in proportion to *n* because rising *n* causes the media movement to become more violent, resulting in a higher *E*.

As shown in Figure 11b, the response surface changes more steeply along the α direction when *d* is 2400 mm, indicating that the effect of α on *E* is more significant, which is because the vortex phenomenon causes an invalid collision, and the maximum value of *E* is obtained when α is minimum and *n* is maximum.

**Figure 12:**
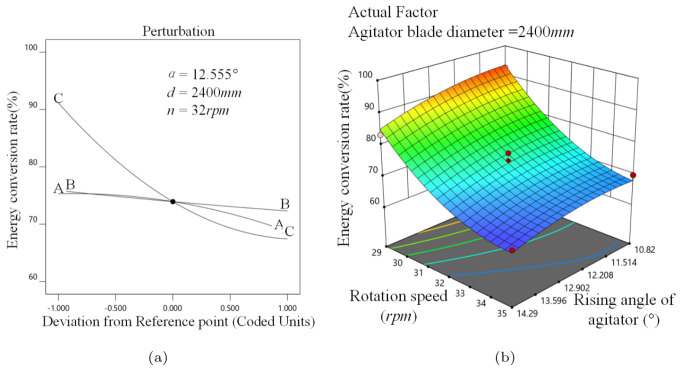
Energy efficiency analysis, (**a**) α, *d*, *n* perturbation effect on *R*, (**b**) α−n interaction on *R*.

Figure 12a demonstrates that *R* reduces as α, *d*, and *n* increase, which is due to a growth in the vortex media. *R* drops as *n* increases because *P* increases more rapidly.

When *d* is 2400 mm, the response surface changes more sharply along the *n* direction, implying that the effect of *n* on *R* is more significant, as illustrated in Figure 12b. The response surface is smoother at 32 rpm ≤*n*≤, 35 rpm, and $10.82^{\circ }$ ≤ α ≤ $14.29^{\circ }$, indicating that α has less impact on *R* when the stirred mill is operating at a high speed.

### 4.3. Multi-Objective Optimization

#### 4.3.1. Prediction Model Establishment

From the analysis in Table 5, Table 6, Table 7, Table 8, Table 9 and Table 10, it could be observed that none of the indices had a *p*-value of greater than 0.01. In addition, the coefficient of determination $R^{2}$ is larger than 0.92, and the adequate precision is greater than 10. This means that any of the aforementioned models can be applied to explore the design space. Now, a prediction model of each collision characteristic indicator following previous equations can be established based on the related ANOVA results.

(26) $$\begin{array}{cc} F_{T}= & 6.82188-0.166809\alpha -0.003227d-0.126229n+\\ \; & 0.000018\alpha d-0.000316\alpha n+0.0000099dn+\\ \; & 0.005087\alpha^{2}+0.00000059d^{2}+0.001928n^{2} \end{array}$$

(27) $$\begin{array}{c} F_{N}=-23.77905-1.37262\alpha +0.032997d-0.200702n+\\ 0.000476\alpha d+0.004279\alpha n-0.000121dn+0.001074\alpha^{2}-\\ 7.44482E-06d^{2}+0.006559n^{2} \end{array}$$

(28) $$\begin{array}{c} f=-6.99913E+09+7.53759E+07\alpha +4.47695E+\\ 06d+7.19176E+07n-1921.22959\alpha d-\\ 7.20461E+05\alpha n-5000.00000dn-1.79389E+\\ 06\alpha^{2}-884.44444d^{2}-5.72222E+05n^{2} \end{array}$$

(29) $$\begin{array}{c} P=-4026.83518-35.62418\alpha +2.1366d+\\ 105.87706n-0.0044\alpha d-2.76369\alpha n+\\ 0.000167dn+4.34453\alpha^{2}-0.000427d^{2}-0.525778n^{2} \end{array}$$

(30) $$\begin{array}{c} E=-3.98970E+05-33000.99213\alpha +1410.51066d-\\ 42141.49328n+8.83958\alpha d-1240.00961\alpha n-\\ 0.851111dn+946.24987\alpha^{2}-0.317637d^{2}+1094.38056n^{2} \end{array}$$

(31) $$\begin{array}{c} R=+1030.15199+3.05494\alpha -0.108579d-\\ 47.2358n+0.002034\alpha d+0.186542\alpha n+0.001169dn-\\ 0.628590\alpha^{2}+7.03360E-06d^{2}+0.595851n^{2} \end{array}$$

**Figure 13:**
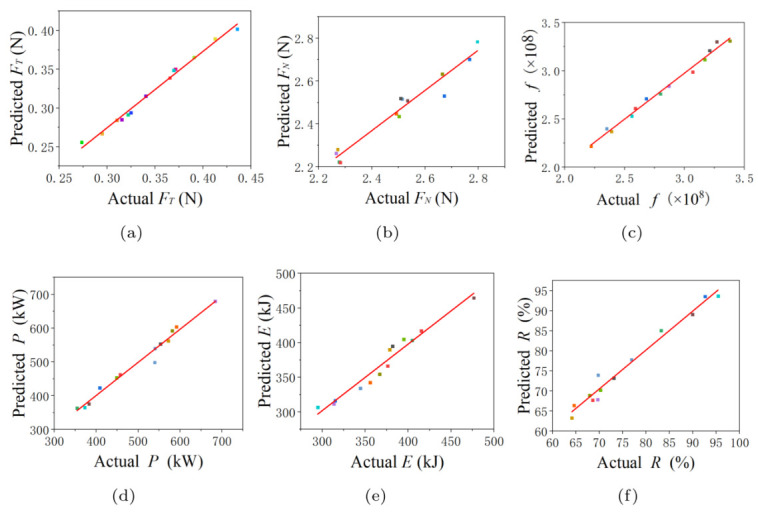
Comparisons between the predicted values and the simulated results. (**a**) Scatter diagram of $F_{T}$, (**b**) Scatter diagram of $F_{N}$, (**c**) Scatter diagram of *f*, (**d**) Scatter diagram of *P*, (**e**) Scatter diagram of *E*, (**f**) Scatter diagram of *R*.

The relationship between the computational results and predicted values is given in Figure 13. It can be seen that the quadratic polynomial model is close to the diagonal, indicating that the predicted values approach the simulated results. Therefore, the model has a good ability for prediction.

#### 4.3.2. Desirability Function Approach

To gain the optimal parameters under multiple objectives, a multi-objective optimization design (MOD) has been employed. The MOD for the VSM performance under various parameters can be expressed as

(32) $$\left\{\begin{array}{c} \;\mathrm{Maximize}\;f_{1}=F_{T}\left(\alpha ,d,n\right)\\ \;\mathrm{Maximize}\;f_{2}=F_{N}\left(\alpha ,d,n\right)\\ \;\mathrm{Maximize}\;f_{3}=f\left(\alpha ,d,n\right)\\ \;\mathrm{Minimize}\;f_{4}=P\left(\alpha ,d,n\right)\\ \;\mathrm{Maximize}\;f_{5}=E\left(\alpha ,d,n\right)\\ \;\mathrm{Maximize}\;f_{6}=R\left(\alpha ,d,n\right)\\ 10.82^{\circ }\leq \alpha \leq 14.29^{\circ }\\ 2250\;\mathrm{mm}\leq d\leq 2550\;\mathrm{mm}\\ 29\;\mathrm{rpm}\leq n\leq 35\;\mathrm{rpm} \end{array}\right.$$

It is necessary to first process each response indicator value into a dimensionless combination parameter. If an objective function is to be maximized, the individual desirability function ($x_{i}$) can be defined by

(33) $$x_{i}=\left\{\begin{array}{cc} 0 & K_{i}\leq L_{i}\\ {\left(\frac{K_{i}-L_{i}}{H_{i}-L_{i}}\right)}^{wt_{i}} & L_{i}<K_{i}<H_{i}\\ 1 & K_{i}\geq H_{i} \end{array}\right.$$

If an objective function is to be minimized, the individual desirability function ($x_{i}$) can be defined by

(34) $$x_{i}=\left\{\begin{array}{cc} 1 & K_{i}\leq L_{i}\\ {\left(\frac{H_{i}-K_{i}}{H_{i}-L_{i}}\right)}^{wt_{i}} & L_{i}<K_{i}<H_{i}\\ 0 & K_{i}\geq H_{i} \end{array}\right.$$

where $K_{i}$ is the response value, $Low_{i}$ represents the lower limit, $High_{i}$ denotes the upper limit, $wt_{i}$ represents the weight factor.

The desirability objective function *D* is combined with these determined individual $x_{i}$ values, and it is a geometric mean of all the $x_{i}$ values.

(35) $$D={\left(\prod_{i=1}^{n}x_{i}^{r_{i}}\right)}^{1/n}$$

where *n* denotes the number of responses, and each response can be given an importance indicator ($r_{i}$) relative to other responses. Importance ($r_{i}$) varies from the least important values 1(+) to the most important value of 5(+++++).

#### 4.3.3. Design Optimization Results

The goal of multi-objective design is to achieve maximum $F_{T}$, maximum $F_{N}$, maximum *f*, maximum *E*, maximum *R*, and minimum *P*. Employing the desirability method, the MOD for grinding performance under various parameters could be formulated as

(36) $$\left\{\begin{array}{cc} \;\mathrm{Maximize}\; & D={\left(x_{F_{T}}\times x_{F_{N}}\times x_{f}\times x_{P}\times x_{E}\times x_{R}\right)}^{1/6}\\ \;S.t.\; & 10.82^{\circ }\leq \alpha \leq 14.29^{\circ }\\ \; & 2250\;\mathrm{mm}\leq d\leq 2550\;\mathrm{mm}\\ \; & 29\;\mathrm{rpm}\leq n\leq 35\;\mathrm{rpm} \end{array}\right.$$

where $x_{F_{T}}$, $x_{F_{N}}$, $x_{f}$, $x_{P}$, $x_{E}$, $x_{R}$ are calculated as in the equations above.

According to the importance of the six indicators, i.e., $x_{F_{T}}$, $x_{F_{N}}$, $x_{f}$, $x_{P}$, $x_{E}$, $x_{R}$, their weights are +, +, +, ++, +++, +++, +++, and the optimal solution results are shown in Table 11. It can be found that the overall composite desirability value of 0.559 is obtained when α = 10.82 °, *d* = 2409.151 mm, and *n* = 29.175 rpm.

Compared with the initial results, the optimized values have improved as shown in Table 12. The power consumption reduces by 8.09% and the collision energy increases by 9.53% with the energy conversion raised by 20.7%. Meanwhile, both the collision strength and frequency between media have been strengthened. In summary, the grinding performance has been enhanced.

## 5. Conclusions

In this work, the DEM has been utilized to simulate the colliding behaviors of grinding balls in an industry-scale VSM. A set of relatively comprehensive indicators has been established to evaluate the grinding performance. Three main parameters, i.e., the stirrer helix angle (α), the mill diameter (*d*), and the rotating speed (*n*) have been focused on and systematically investigated. The grinding performances under different conditions have been analyzed by RSM (response surface method). Moreover, based on multi-objective optimization design, the three arguments have been optimized, which could establish a whole understanding of the grinding performance and give instructions for the design, running, and optimization of large-scale VSMs. The main conclusions are as follows:

(1) For a large-scale dry vertical stirred mill, an eddy current phenomenon has been discovered, which is the vortex media near the stirring shaft. It is found that the number of vortex media grows with α or *d* but decreases with *n*. This is not emphasized in previous investigations.
(2) A drop in α or an increase in *d* will result in an increase in *P* (power consumption). Then, $F_{N}$ (normal collision force) lowers as *n* grows. As *d* grows, *f* (collision frequency) rises and then falls. In addition, *E* (collision energy) diminishes when α or *d* rises.
(3) The rotating speed (*n*) of the stirrer has great importance for the VSM performance. When the mill is running at a low speed, the effect of *d* on *f* will be dominant. In addition, when the mill is running speedily, *R* (energy conversion rate) is less affected by the agitator structure.
(4) A prediction model of the media collision performance is established, and it is in good agreement with the computational results. On this basis, the important parameters are optimized, resulting in the increase of *R* and *E* values by 20.7% and 9.53%, respectively, and the decrease of *P* value by 8.09%. Additionally, the collision intensity and collision frequency were also enhanced.

## Figures and Tables

**Table 1:** Initial structural parameters.

Parameter	*H* (mm)	*h* (mm)	*D* (mm)	*d* (mm)	α ${(}^{\circ })$
Value	4300	3650	2900	2400	13.61

**Table 2:** The numerical simulation conditions in this work.

Parameters	Value
Corundum Density (kg/m${}^{3}$)	3900
Steel Density (kg/m${}^{3}$)	7850
Corundum Shear modulus (10${}^{9}$ Pa)	0.1
Steel Shear modulus (10${}^{9}$ Pa)	80
Corundum $Poisson^{\prime }sratio$	0.25
Steel $Poisson^{\prime }sratio$	0.25
Media—media restitution coefficient	0.3
Media—cylinder wall restitution coefficient	0.3
Media—screw agitator wall restitution coefficient	0.3
Media—media sliding friction	0.15
Media—cylinder wall sliding friction	0.21
Media—screw agitator wall sliding friction	0.21
Media—media rolling friction	0.01
Media—cylinder wall rolling friction	0.01
Media—screw agitator wall rolling friction	0.01
Total mass of media (kg)	35,500
Media gradation	M (ϕ15 mm): M (ϕ22 mm) = 1:1
Stirrer speed (rpm)	32
Recorded simulation time (s)	15

**Table 3:** The simulated changing values of the influencing factors.

Parameters	Code	Lower (−1)	Mean (0)	Upper (1)
Helix angle (${}^{\circ }$)	α	10.82	12.55	14.29
Diameter (mm)	*d*	2250	2400	2550
Rotation Speed (rpm)	*n*	29	32	35

**Table 4:** Design experiment schemes and response values.

CaseNo.	α ${(}^{\circ })$	*d* (mm)	*n* (rpm)	$F_{T}$ (N)	$F_{N}$ (N)	*f* $\left(\times 10^{8}\right)$	*P* (kW)	*E* (J/S)	*R* (%)
1	10.82	2250	32	0.3402	2.7669	2.59	540.1	415,253	76.879
2	14.29	2250	32	0.3153	2.2773	2.68	448.5	315,026	70.240
3	10.82	2550	32	0.3711	2.2654	2.80	553.4	404,609	73.119
4	14.29	2550	32	0.3654	2.2714	2.87	457.1	313,584	68.597
5	10.82	2400	29	0.3103	2.7964	2.39	408.6	378,475	92.634
6	14.29	2400	29	0.2948	2.5339	2.57	354.1	294,730	83.236
7	10.82	2400	35	0.4126	2.6651	3.27	684.0	476,407	69.648
8	14.29	2400	35	0.3905	2.4917	3.30	572.0	366,845	64.134
9	12.555	2250	29	0.2734	2.5135	2.22	372.7	355,619	95.407
10	12.555	2550	29	0.3218	2.5026	2.35	382.7	344,188	89.927
11	12.555	2250	35	0.3694	2.5081	3.17	581.1	394,816	67.948
12	12.555	2550	35	0.4356	2.2811	3.21	591.4	381,853	64.572
13	12.555	2400	32	0.3248	2.6723	3.07	539.4	376,164	69.740
14	12.555	2400	32	0.3046	2.4762	3.06	498.4	370,475	74.328
15	12.555	2400	32	0.3226	2.5742	3.03	495.6	369,502	74.551
16	12.555	2400	32	0.3124	2.5252	2.89	485.6	359,096	73.943
17	12.555	2400	32	0.3326	2.5497	2.89	462.5	356,845	77.151

**Table 5:** Analysis of variance for $F_{T}$.

Source	Sum of Squares	Df	Mean Square	F-Value	*p*-Value
Model	$5.81\times 10^{4}$	9	0.0033	35.7355	<0.0001
α	$4.78\times 10^{3}$	1	0.0006	6.2963	0.0404
*d*	0.02	1	0.0048	51.8628	0.0002
*n*	$9.22\times 10^{5}$	1	0.0208	225.1829	<0.0001
a−d	$1.08\times 10^{5}$	1	0.0001	0.9989	0.3509
a−n	$7.97\times 10^{5}$	1	0.0000	0.1170	0.7424
d−n	$9.87\times 10^{4}$	1	0.0001	0.8634	0.3837
$A^{2}$	$7.47\times 10^{4}$	1	0.0010	10.7008	0.0137
$d^{2}$	$1.27\times 10^{3}$	1	0.0007	8.0941	0.0249
$n^{2}$	$6.46\times 10^{4}$	1	0.0013	13.7379	0.0076
Residual	$1.65\times 10^{4}$	7	0.0001		
Lack of Fit	$4.81\times 10^{4}$	3	0.0001	0.4578	0.7265
Pure Error	0.03	4	0.0001		
Cor Total	$5.81\times 10^{4}$	16			
$R^{2}$	Adjusted $R^{2}$	Predicted $R^{2}$	Adequate precision		
0.9786	0.9513	0.8881	20.474		

**Table 6:** Analysis of variance for $F_{N}$.

Source	Sum of Squares	Df	Mean Square	F-Value	*p*-Value
Model	0.3995	9	0.0444	10.0783	0.0030
α	0.1057	1	0.1057	24.0020	0.0018
*d*	0.0696	1	0.0696	15.8118	0.0053
*n*	0.0201	1	0.0201	4.5749	0.0697
a−d	0.0614	1	0.0614	13.9394	0.0073
a−n	0.0020	1	0.0020	0.4505	0.5236
d−n	0.0118	1	0.0118	2.6766	0.1458
$A^{2}$	0.0000	1	0.0000	0.0100	0.9232
$d^{2}$	0.1181	1	0.1181	26.8249	0.0013
$n^{2}$	0.0147	1	0.0147	3.3315	0.1107
Residual	0.0308	7	0.0044		
Lack of Fit	0.0097	3	0.0032	0.6092	0.6436
Pure Error	0.0212	4	0.0053		
Cor Total	0.4303	16			
$R^{2}$	Adjusted $R^{2}$	Predicted $R^{2}$	Adequate precision		
0.9283	0.8362	0.7636	10.9921		

**Table 7:** Analysis of variance for *f*.

Source	Sum of Squares	Df	Mean Square	F-Value	*p*-Value
Model	$1.73\times 10^{16}$	9	$1.92\times 10^{15}$	30.0317	<0.0001
α	$1.71\times 10^{14}$	1	$1.71\times 10^{14}$	2.6735	0.1460
*d*	$4.06\times 10^{14}$	1	$4.06\times 10^{14}$	6.3450	0.0399
*n*	$1.46\times 10^{16}$	1	$1.46\times 10^{16}$	228.4198	<0.0001
a−d	$1\times 10^{12}$	1	$1\times 10^{12}$	0.0156	0.9040
a−n	$5.625\times 10^{13}$	1	$5.625\times 10^{13}$	0.8788	0.3797
d−n	$2.025\times 10^{13}$	1	$2.025\times 10^{13}$	0.3164	0.5913
$A^{2}$	$1.23\times 10^{14}$	1	$1.23\times 10^{14}$	1.9182	0.2086
$d^{2}$	$1.67\times 10^{15}$	1	$1.67\times 10^{15}$	26.0504	0.0014
$n^{2}$	$1.12\times 10^{14}$	1	$1.12\times 10^{14}$	1.7447	0.2281
Residual	$4.48\times 10^{14}$	7	$6.40\times 10^{13}$		
Lack of Fit	$1.19\times 10^{14}$	3	$3.975\times 10^{13}$	0.4836	0.7115
Pure Error	$3.29\times 10^{14}$	4	$8.22\times 10^{13}$		
Cor Total	$1.77\times 10^{16}$	16			
$R^{2}$	Adjusted $R^{2}$	Predicted $R^{2}$	Adequate precision		
0.9748	0.9423	0.8635	17.9675		

**Table 8:** Analysis of variance for *P*.

Source	Sum of Squares	Df	Mean Square	F-Value	*p*-Value
Model	$1.215\times 10^{5}$	9	13497.37	24.21	0.0002
α	$1.569\times 10^{4}$	1	15,696.38	28.15	0.0011
*d*	222.1832	1	222.18	0.3986	0.5479
*n*	$1.036\times 10^{5}$	1	$1.036\times 10^{5}$	185.81	<0.0001
a−d	5.24	1	5.24	0.0094	0.9255
a−n	827.71	1	827.71	1.48	0.2625
d−n	0.0225	1	0.0225	0.0000	0.9951
$A^{2}$	720.14	1	720.14	1.29	0.2931
$d^{2}$	389.42	1	389.42	0.6986	0.4309
$n^{2}$	94.28	1	94.28	0.1691	0.6932
Residual	3902.24	7	557.46		
Lack of Fit	787.34	3	262.45	0.3370	0.8009
Pure Error	3114.90	4	778.73		
Cor Total	$1.254\times 10^{5}$	16			
$R^{2}$	Adjusted $R^{2}$	Predicted $R^{2}$	Adequate precision		
0.9689	0.9289	0.8607	17.4594		

**Table 9:** Analysis of variance for *E*.

Source	Sum of Squares	Df	Mean Square	F-Value	*p*-Value
Model	$2.709\times 10^{10}$	9	$3.0103\times 10^{9}$	15.3689	0.0008
α	$1.849\times 10^{10}$	1	$1.8486\times 10^{10}$	94.3765	<0.0001
*d*	$1.663\times 10^{8}$	1	$1.6635\times 10^{8}$	0.8493	0.3874
*n*	$7.621\times 10^{9}$	1	$7.6205\times 10^{9}$	38.9056	0.0004
α−d	$2.117\times 10^{7}$	1	$2.1169\times 10^{7}$	0.1081	0.7520
α−n	$1.666\times 10^{8}$	1	$1.6663\times 10^{8}$	0.8507	0.3870
d−n	$5.868\times 10^{5}$	1	$5.8676\times 10^{5}$	0.0030	0.9579
$\alpha^{2}$	$3.416\times 10^{7}$	1	$3.4162\times 10^{7}$	0.1744	0.6887
$d^{2}$	$2.151\times 10^{8}$	1	$2.1506\times 10^{8}$	1.0980	0.3295
$n^{2}$	$4.085\times 10^{8}$	1	$4.0847\times 10^{8}$	2.0854	0.1919
Residual	$1.371\times 10^{9}$	7	$1.9587\times 10^{8}$		
Lack of Fit	$1.105\times 10^{9}$	3	$3.6830\times 10^{8}$	5.5340	0.0659
Pure Error	$2.662\times 10^{8}$	4	$6.6552\times 10^{7}$		
Cor Total	$2.846\times 10^{10}$	16			
$R^{2}$	Adjusted $R^{2}$	Predicted $R^{2}$	Adequate precision		
0.9518	0.8899	0.7643	14.7072		

**Table 10:** Analysis of variance for *R*.

Source	Sum of Squares	Df	Mean Square	F-Value	*p*-Value
Model	1374.75	9	152.75	23.19	0.0002
α	84.98	1	84.98	12.90	0.0088
*d*	25.41	1	25.41	3.86	0.0902
*n*	1125.81	1	1125.81	170.94	<0.0001
α−d	1.12	1	1.12	0.1702	0.6923
α−n	3.77	1	3.77	0.5726	0.4739
d−n	1.11	1	1.11	0.1680	0.6941
$\alpha^{2}$	15.08	1	15.08	2.29	0.1741
$d^{2}$	0.1055	1	0.1055	0.0160	0.9029
$n^{2}$	121.09	1	121.09	18.39	0.0036
Residual	46.10	7	6.59		
Lack of Fit	17.63	3	5.88	0.8257	0.5444
Pure Error	28.47	4	7.12		
Cor Total	1420.85	16			
$R^{2}$	Adjusted $R^{2}$	Predicted $R^{2}$	Adequate precision		
0.9676	0.9258	0.7702	15.4310		

**Table 11:** The multi-objective optimization results.

Parameter	α ${(}^{\circ })$	*d* (mm)	*n* (rpm)	Composite Desirability
Value	10.82	2409.151	29.175	0.559

**Table 12:** Comparison between the initial and optimized results.

Parameter	$F_{T}$ (N)	$F_{N}$ (N)	*f* (106)	*P* (kW)	*E* (kJ)	*R* (%)
Original	0.310	2.525	239.1	485.6	359.1	73.94
Optimized	0.312	2.772	247.7	446.3	393.3	89.25
Growth	0.65%	9.77%	3.59%	−8.09%	9.53%	20.70%

## Data Availability

Data available on request from the corresponding author.

## Nomenclature

α	stirrer helix angle	${}^{\circ }$
*d*	stirrer diameter	mm
*n*	rotating speed	rpm
$F_{T}$	tangential collision force	N
$F_{N}$	normal collision force	N
*f*	collision frequency	s${}^{-1}$
*P*	stirrer power	kW
*E*	collision energy	J/S
*R*	energy conversion rate	−
$F_{\phi 15-\phi 15}$	average collision force between ϕ15 mm media	N
$F_{\phi 15-\phi 22}$	average collision force between ϕ15 mm and ϕ22 mm media	N
$F_{\phi 22-\phi 22}$	average collision force between ϕ22 mm media	N
Δt	saving time interval	s
$n_{i}$	total number of collisions of media	1
$n_{\phi 15-\phi 15}$	number of collisions between ϕ15 mm media	1
$n_{\phi 15-\phi 22}$	number of collisions between ϕ15 mm and ϕ22 mm media	1
$n_{\phi 22-\phi 22}$	number of collisions between ϕ22 mm media	1
$\bar{n}$	average total number of collisions	1
$N_{t}$	number of nodes	1
$\bar{T}$	average torque at different moments	N m
$T_{i}$	stirrer torque	N m
$N_{t}$	number of nodes	N m
$E_{i}$	total collision energy loss	J
$E_{\phi 15-\phi 15}$	collision energy loss between ϕ15 mm media	J
$E_{\phi 15-\phi 22}$	collision energy loss between ϕ15 mm and ϕ22 mm media	J
$E_{\phi 22-\phi 22}$	collision energy loss between ϕ22 mm media	J
$\bar{E}$	average total collision energy loss	J
*m*	mass	kg
*v*	velocity	m/s
*w*	angular velocity	rad/s
*I*	rotational inertia	kg m${}^{2}$
$F_{n,ji}$	normal forces between balls *i* and *j*	N
$F_{t,ij}$	tangential forces between balls *i* and *j*	N
*g*	gravity acceleration	m s${}^{-2}$
$R_{i}$	radius of ball *i*	m
*t*	time	s
$\delta_{n}$	normal overlap of the two balls	m
$\delta_{t}$	tangential overlap of the two balls	m
$v_{n,ij}$	normal velocities between the two balls	m/s
$v_{t,ij}$	tangential velocities between the two balls	m/s
$\mu_{s,ij}$	static friction coefficient	−
$E^{*}$	equivalent elastic modulus	Pa
$R^{*}$	equivalent contact radius	m
$G^{*}$	equivalent shear modulus	Pa
$m^{*}$	equivalent mass of the two balls	kg
$E_{k}$	Young’s modulus	Pa
$m^{*}$	mass	kg
$u_{k}$	Poisson’s ratio of balls	−
*H*	cylinder height	mm
*h*	screw agitator height	mm
*D*	cylinder diameter	mm
